# Stochastic accumulation of feature information in perception and memory

**DOI:** 10.3389/fpsyg.2014.00412

**Published:** 2014-05-12

**Authors:** Christopher Kent, Duncan Guest, James S. Adelman, Koen Lamberts

**Affiliations:** ^1^Bristol Tactile Action and Perception Lab, School of Experimental Psychology, University of BristolBristol, UK; ^2^Division of Psychology, School of Social Sciences, Nottingham Trent UniversityNottingham, UK; ^3^Department of Psychology, University of WarwickCoventry, UK; ^4^Vice-Chancellor’s Department, University of YorkYork, UK

**Keywords:** information accumulation, perception, memory, cognition, categorization, identification, visual search, reading

## Abstract

It is now well established that the time course of perceptual processing influences the first second or so of performance in a wide variety of cognitive tasks. Over the last 20 years, there has been a shift from modeling the speed at which a display is processed, to modeling the speed at which different features of the display are perceived and formalizing how this perceptual information is used in decision making. The first of these models (Lamberts, 1995) was implemented to fit the time course of performance in a speeded perceptual categorization task and assumed a simple stochastic accumulation of feature information. Subsequently, similar approaches have been used to model performance in a range of cognitive tasks including identification, absolute identification, perceptual matching, recognition, visual search, and word processing, again assuming a simple stochastic accumulation of feature information from both the stimulus and representations held in memory. These models are typically fit to data from signal-to-respond experiments whereby the effects of stimulus exposure duration on performance are examined, but response times (RTs) and RT distributions have also been modeled. In this article, we review this approach and explore the insights it has provided about the interplay between perceptual processing, memory retrieval, and decision making in a variety of tasks. In so doing, we highlight how such approaches can continue to usefully contribute to our understanding of cognition.

## INTRODUCTION

A growing body of evidence, using detailed mathematical models of the time course of perception and memory, clearly demonstrates that information about a stimulus in the environment and about memory representations becomes gradually available over time (e.g., Purcell et al., 2010). Over the last 20 years models of perceptual information accumulation have begun to be integrated within formal models of cognitive processes based on the principle that, in everyday life, we frequently have to make very quick decisions about what objects are and what properties they have (e.g., Lamberts, 2002). For example, in the laboratory it might take 1,000 ms to categorize a stimulus correctly. This time will partly be determined by the time to make a decision and the time to produce a response, but a significant proportion of the time will involve perceptual processing, i.e., the time taken to accumulate object information and form a representation of the object. In everyday life, we might have much less time to make a decision considering that we make 3–4 fixations per second in a dynamic visual scene that changes as we move through it. This means that we often make decisions based on incomplete perceptual information, either because the amount of perceptual processing time is limited or because of environmental conditions such as occlusion or poor lighting. Similarly, many cognitive tasks require the reconstruction, from memory, of past stimuli, and there may be insufficient time for this reconstructive retrieval process to be completed. It is therefore important to understand how stimulus information is accumulated from both perception and memory, integrated and utilized in cognitive tasks, and the extent to which errors in such tasks can be attributable to making decisions based on incomplete perceptual and memorial representations.

In this article we argue that stochastic sampling of feature information is a process common to both perception and memory and that it underlies early performance in a number of tasks including categorization and identification, recognition and matching, visual search, and word identification. Our literature review centers on the development of a stochastic feature sampling process first instantiated in a model by Lamberts (1995). While this focus means our review is not exhaustive, it allows us to provide a comprehensive account of one of the most broadly applied approaches to integrating a formalized feature sampling mechanism with models of cognition. We show that by embodying relatively simple central concepts the model can help us explore the similarities and difference across tasks, which other, more specific, approaches may miss. We end with some thoughts on the future directions for the field and the inclusion of stochastic sampling processes in models of memory and perception more broadly.

## STOCHASTIC INFORMATION ACCUMULATION

Our approach to modeling the time course of perception and retrieval relies on the process of sampling information elements from either a physical stimulus or a memorial representation of a previous stimulus. We do not attempt a complete history of the field here, but instead note that current ideas about the nature of stimulus perception can be traced most fully back to the seminal *Psychological Review* article by Estes (1950) and his statistical theory of learning. Not only did stimulus sampling theory kick-start a major shift toward the development of formal mathematical models of learning and memory (e.g., LaBerge, 1959; Bower, 1967; McGill, 1967; Norman and Rumelhart, 1970; Rumelhart, 1970; Wolford, 1975; amongst many others), but it highlighted the importance of variability in stimulus perception and the stochastic nature of stimulus sampling (see Bower, 1994, for a review of the impact of Estes, 1950). According to the stimulus sampling process (see Estes and Burke, 1953, for a detailed account), a stimulus consists of a finite population of small independent elements that are sampled randomly. Because the process is stochastic, the number of sampled elements varies between presentations. The currently perceived stimulus is therefore the average number of elements sampled within the duration of presentation. Each element is associated, through learning, with a response, such that element sampling gradually builds up evidence for one response over other responses. This simple conception has had a dramatic impact on the field of cognitive psychology (see Bower, 1994, and the two volumes edited by Healy et al., 1992a,b) and continues to inspire models of perception and memory. For example, sequential sampling models often include the assumption that the decision process is driven by perceptual sampling, e.g., see, Townsend and Ashby, 1983; Luce, 1986; Usher and McClelland, 2001; Brown and Heathcote, 2005; Purcell et al., 2010, for reviews). An alternative to the popular sequential sampling models is to assume participants continue accumulating information until they have sufficient evidence for one response over another, based on the Luce choice rule (Luce, 1963), this is the option we adopt, as it allows a detailed examination of the time course of object-feature information accumulation (Lamberts, 2000).

Estes’s conception of stochastic sampling of elements is still at the heart of the theoretical approach of the core models we review here. However, the exact meaning of an element is somewhat different from one where elements might represent whole items (e.g., letters or digits) but instead refers to subcomponents of a stimulus feature (e.g., Bower, 1967; Rumelhart, 1970). It is therefore important to outline explicitly what we mean by an element. An element is a hypothetical construct relating to a basic unit of information about a stimulus. Features are composed of a number of non-differentiated elements. For mathematical tractability, it can often be assumed that a single element (for binary valued dimensions) or a small number of elements (for continuously valued dimensions) are available for sampling without a great loss of precision. Elements are sampled from a sensory store in order to establish a stimulus representation in (for example) visual short term memory (VSTM) upon which a perceptual decision can be made. This conception is common to many other current models (see Purcell et al., 2010, for a discussion of the different types). Critically, however, the approach we take models accumulation of individual object features, whereas most other models focus on the stimulus as a whole. Thus the model is able to account for how an incomplete stimulus representation drives perceptual decisions. Sampling of elements is: random without replacement; discrete, in that partial information about an element cannot exist; and the probability of sampling one element is independent of sampling any other element. These properties result in a Poisson process, the time between events in a Poisson process is described by an exponential distribution. Thus the cumulative probability of a feature *x* being included at or before time *t* is

$$i_{x}(t)=\lambda \left[1-e^{-\beta_{x}\left(t-\delta \right)}\right]$$

in which λ is the asymptotic probability of a feature being included (which may be less than 1 for noisy stimuli or weakly encoded representations), β is the rate at which the information is sampled about that feature, and δ is any non-processing time (e.g., decision and motor response times). This simple equation can be used to model the information accumulation process for each feature from both an external stimulus, and from memory. Different tasks will require different decision rules, but the strong argument which we attempt to demonstrate in the following literature review is that the general form of the information accumulation process remains fixed across tasks.

## ESTIMATING ACCUMULATED INFORMATION

Although the notion of stochastic information accumulation is employed in many models, directly estimating the amount of information accumulated and utilized in a cognitive task is difficult. Many experiments on early stimulus perception involved (and continue to involve) presenting a stimulus for a brief period and then measuring the time to respond (RT). Although useful for many purposes, using RTs as a dependent measure, however, can confound processing time (forcing a quick response) with the time of stimulus exposure (brief stimulus durations; Wickelgren, 1977). A problem arises because participants are able to trade accuracy for speed. Participants may choose to respond to a briefly presented stimulus quickly but sacrifice accuracy, or take more time to reach a higher level of accuracy. This speed accuracy trade-off (SAT) means that differences in accuracy at short lags, or differences in RTs at longer lags may represent strategic shifts by the participants. Wickelgren (1977, see also Pachella, 1974; Wickelgren, 1975; Dosher, 1976, 1979) strongly argued against the use of only RT data from free-RT experiments to test models about the dynamics of cognition. This is especially true when error rates are very low as these are the conditions in which large variations in RTs may be seen for very small changes in accuracy between conditions (which might not be observable with even large sample sizes), which is typically where free-RTs are most often used. A single mean RT is therefore not very helpful in evaluating theories about the time course of information accumulation. Models need to predict both accuracy and RTs for various levels of performance, since a given RT could be achieved by a participant setting a certain level of accuracy. Control of the SAT therefore needs to be taken away from the participants and built into the experimental design. Means of achieving such control focus on the idea of a signal-to-respond (STR) paradigm in which participants are cued to respond after different intervals after the onset of a stimulus. Examining performance at very short signal lags through to longer signal lags enables a picture of the time course of performance to be built up that can be used to estimate how information is accumulated.

Reed (1973, 1976) used the offset of the stimulus to cue when to respond and mixed response signal conditions within block so that participants would not know ahead of the signal how much time they would have to respond, reducing the possibility of strategic control (although see Heit et al., 2003, 2008, who demonstrate that participants can shift their strategic responding even under STR conditions). A further refinement of the method was proposed by Meyer et al. (1988) who argued that tasks involving only short signal trials meant that participants may employ a completely different strategy to the one they would adopt under free-RT. They proposed that free-RT trials should also be included in the mix of signal trials, and outlined a detailed method (the titrated reaction time procedure) and analysis for speed-accuracy decomposition. With these concerns in mind, modern application of the STR procedure typically includes a number of longer response times to ensure “normal” strategic processing is used. However, the decomposition technique suggested by Meyer et al. (1988) is most relevant to situations in which accuracy grows monotonically over the time course of a trial. As the literature review will make clear, this is not always the case, and non-monotonic SAT curves are particularly informative about the piecemeal nature of stimulus processing.

## MODELING SAT CURVES

Modeling SAT curves yields useful information about the processing dynamics of a task. There are two broad approaches in modeling these SAT curves. In one approach, the one we refer to above, an assumption about information accumulation about a stimulus or its features is built in to a process model of the cognitive task, that is, a model that indicates how this information is utilized within that given task. An example of this is perceptual categorization, whereby a participant accumulates information about the stimulus, building up a stimulus representation that is then used to determine which category the stimulus belongs to. Information accumulation thus has to be integrated with a model about exactly how that perceptual information is used to make a decision. An alternative approach is to focus only on the dynamics of information accumulation and to examine how these differ between experimental conditions or between participants. In this approach, the SAT curve is produced for a given condition, and then a shifted exponential rise to asymptote function is fit to this curve (empirically the exponential function fits well). The exponential function is similar to that in the equation above, except that this does not refer to the probability of feature inclusion, but a measure of task performance. This form of the STR method and analysis has been influential and is important in several respects. First, it has been used widely throughout cognitive psychology in order to explore the time course of information accumulation estimates from the processing dynamics of different tasks. Second, the models of cognition that this review focuses on have utilized this formalization of information accumulation and integrated it within models describing cognitive tasks. Third, the strongest tests of these models necessarily come from experiments using the STR methodology, as these provide critical information about object-feature accumulation. We now consider how this simple formulation of information accumulation, which has provided the core processing assumption of various related models, is useful in comparing the similarities between several important cognitive tasks.

## IDENTIFICATION AND CATEGORIZATION

In many categorization studies, the participant’s job is to learn to associate *n* stimuli with *m* response options (*m* < *n*). The *n* stimuli are typically composed of a number of either binary or continuously valued feature dimensions. Identification is a special case of categorization in which *n* = *m*. Absolute identification is a special case of identification in which stimuli vary along only a single dimension (e.g., loudness or brightness). Because of these simple relationships, it is likely that the three tasks involve the same underlying processes and only minimal adjustments to the decision rules should be necessary for a model of one task to generalize to the other tasks. Although multiple models of these tasks have been developed, we focus in one in particular, the extended generalized context model (EGCM; Lamberts, 1995, 1998, 2000) that highlights the role of a perceptual information accumulation process in determining performance in these tasks.

The EGCM is a development of the GCM (Nosofsky, 1986) in which a decision to categorize a stimulus into one category or another is based on the summed similarity of that stimulus to the stored members (exemplars) of each of the categories (using the Luce, 1963, choice ratio). Stimuli are represented as points in a psychological space, the dimensions of which correspond to different features. Similarity between stimuli is a decreasing function – the generalization gradient – of their distance in this psychological space (Shepard, 1957). Of central importance to the GCM is that this psychological space is malleable. A dimension can be weighted more strongly if it is important for successful categorization such that distances along this dimension in psychological space are stretched. This utility weighting is limited in capacity such that increasing the utility weight allocated to one dimension decreases the utility of another. This acts to stretch and shrink the psychological space thus making similarity more or less dependent on different dimensions.

Using a categorization task in which a stimulus was presented for different durations and then had to be categorized (participants had already been trained to categorize a subset of stimuli) Lamberts (1995) fit the GCM to categorization performance at the different durations and showed that with increasing time to process the stimuli the generalization gradient for categorization becomes steeper and the utility weight distribution changes. That is, the differences between a stimulus and exemplars in memory had a larger impact on categorization with more time to process stimuli. This led Lamberts (1995) to suggest a role for perceptual processing in categorization whereby, early on, when processing time is short, limited information about stimulus features is available from which to make category decisions (the stimulus is undifferentiated along the different dimensions), whereas later on category decisions are based on more complete representation of the stimulus. Importantly, changes in categorization performance over time can be explained by differences in the rate at which stimulus dimensions are processed. For example, if color is processed faster than shape, then categorization decisions in which color information is critical will be accurate earlier than categorization decisions for which shape information is critical. These principles were formalized in the EGCM, in which the similarity between a presented stimulus and stored exemplars (and thus categorization performance) is determined by whether or not perceptual information about each stimulus dimension is available. The formalization of information accumulation is similar to that already given above, with the rate at which perceptual information about a dimension is accumulated thought to reflect the perceptual salience of the stimulus dimension (we return to this issue later).

The validity of the EGCM has been extensively tested by showing it can provide a good fit to data on the time course of performance in a variety categorization tasks in which participants have to learn to categorize a set of stimuli comprised of a number of different dimensions (e.g., line drawings of faces comprised of different mouths, noses, and eyes) and later have to categorize these (and new) stimuli under time pressure (Lamberts, 1995, 1998; Lamberts and Brockdorff, 1997; Lamberts and Freeman, 1999a,b). Of particular importance, Lamberts and Freeman (1999a) clearly demonstrated the importance of perceptual processes in accounting for categorization performance. In one task Lamberts and Freeman (1999a) trained people to categorize objects. Later they asked participants to categorize incomplete objects (where one or more features had been removed). Finally, participants categorized complete objects again, but under time pressure. Lamberts and Freeman (1999a) reasoned that if the EGCM is correct and object representations are built gradually from stochastically sampled features, then they should see a systematic correspondence in category choice early on in processing whole objects under time pressure and part-stimuli under free-RT. Across two experiments the pattern of categorization of part-stimuli closely matched that of whole stimuli categorized at short durations providing strong support for the feature sampling mechanism.

In a second task, Lamberts and Freeman (1999a) used a category structure in which one stimulus in Category A shared many features of stimuli in Category B. They showed that, under limited time pressure, this stimulus tended to be incorrectly categorized early on in processing before being categorized accurately at longer stimulus durations. This category cross over effect is important because other models that do not utilize a feature based perceptual processing component struggle to account for it. That is, if a model assumes similarity is static and time invariant, then it is difficult for it to predict anything other than a monotonic increase from chance level performance with increases in time for categorization. The EGCM can account for this because fast processing of a feature commonly associated with one category will make the stimulus seem more similar to that category when time for processing is short and limited information about other stimulus dimensions is available.

This development and testing of the EGCM indicated the importance of perceptual processing mechanisms in determining performance in categorization and showed how a feature based perceptual processing component could be integrated into a formal model of categorization. Subsequently, this led to another key model of categorization, the exemplar based random walk model (EBRW; Nosofsky and Palmeri, 1997) being extended to incorporate a perceptual processing component so that it could account for the time course of categorization of stimuli with separable-dimensions (Cohen and Nosofsky, 2003).

Given the importance of perceptual processing in categorization, a key question is what determines the rate that a stimulus dimension is processed. Lamberts (1995, 1998) suggested that processing rates were independent of the utility of the dimension (how important a dimension is for correct categorization) and in several experiments demonstrated that perceptual processing rates were determined largely by perceptual salience of the dimension and not dimension utility (changing the category structure had little effect on processing rates; see also Ashby and Maddox, 1994; Maddox and Bogdanov, 2000; Maddox, 2001; Maddox and Dodd, 2003). However, given that visual attention is known to modulate sensory processing (e.g., Luck et al., 1994; Treue, 2001; Carrasco et al., 2002), accelerate the rate of perceptual processing (Carrasco and McElree, 2001), and can be flexibly allocated (Bundesen, 1990), Guest and Lamberts (2010) re-examined conditions under which knowledge of the category structure can influence perceptual processing rates. Their experiments used a categorization task in which all stimulus dimensions needed to be processed in order to ensure correct categorization but where stimulus dimensions clearly differed in their diagnosticity (how diagnostic they were of category membership) and diagnosticity was pitted against the perceptual salience of stimulus dimensions. Under these conditions, Guest and Lamberts (2010) found evidence for prioritization of perceptual processing: diagnosticity accelerated the rate of feature information accumulation. This finding raises questions about the nature of the mechanism responsible for prioritization including whether there might be multiple systems for controlling utility weighting and prioritization.

One of the central assumptions of the feature sampling model encapsulated in the EGCM is that feature information is continually combined and integrated into a percept that is used to access memory. Recent research on rule based categorization has shown that this is not necessarily the case. Fific et al. (2010) examined how processing of different dimensions of a stimulus proceeds when a category set can be defined by a set of rules. In these studies, a category set is defined by two features that can either be spatially separable (as is typical of the types of stimuli to which the EGCM has been applied) or integral (e.g., colors varying in brightness and saturation). Fific et al. (2010) examined whether independent logical rules (such as those employed by decision bound theory, e.g., Ashby and Townsend, 1986; Maddox and Ashby, 1993) for each feature (is feature X larger than a criterion, is feature Y larger than a criterion) are used sequentially or in parallel or whether evidence from both features was combined in order to make a coactive category judgment (as in the EBRW). They found evidence for mostly serial processing of dimensions when the features were spatially separable, a mix of sequential and parallel processing when the features were separable but spatially overlaid (Little et al., 2010), and coactive processing when stimuli were integral (Little et al., 2013). Thus, in contrast to the feature sampling principles of the EGCM, coactive integration of feature information was not apparent using stimuli with separable dimensions. However, the models tested by Fific et al. (2010) did not include the EGCM, which likely falls somewhere in between the models tested. It is likely that the EGCM will not easily mimic the serial use of decision rules observed by Fific et al. (2010) and so some modification to the current model in situations where serial processing is encouraged is likely. For example, the introduction of variable takeoff times (currently included as constants in the residual time parameter) for information accumulation along the different dimensions, or the inclusion of a different decision rule, which encapsulates serial feature comparison between the perceptual information and stored information (either exemplars of decision criteria), may allow the EGCM to account for the apparent serial use of feature information when evaluating rules. Neither of these changes affect the nature of the stochastic sampling process. In addition, we note that during other tasks, such as visual search, there is evidence that overlaid features are combined to form percepts of display objects (Takeda et al., 2007) rather than search proceeding based on independent feature comparisons (Treisman and Gelade, 1980). Thus serial, parallel or coactive processing may depend on task demands.

In light of Fific et al.’s (2010) findings, it is instructive at this stage to consider what processing rates themselves might reflect (we thank a reviewer for this suggestion). Differential feature processing rates might, in some cases, be the result of preferential serial processing of that information. Thus prioritization of feature processing (Guest and Lamberts, 2010) may be the result of a serial processing strategy developed in category learning and based on feature diagnosticity. Indeed, Rehder and Hoffman (2005) demonstrated a clear association between attention weights in categorization models and eye fixations on features. Such a conceptualization offers feature sampling models multiple mechanisms for accounting for and modeling the effects of feature salience and feature validity which are well known to tradeoff (e.g., see Kruschke and Johansen, 1999).

An important development of the EGCM is the EGCM-RT (Lamberts, 2000) which accounts for RTs in free-RT tasks. The EGCM-RT explicitly states that as perceptual information is processed, information elements (subcomponents of features) are sampled (not just whole features). This allows the model to predict a gradual increase in information about a feature. As each element is sampled, the evidence for the different categories is evaluated, based on summed similarity. A decision to either stop perceptual processing and produce a response, or to continue to sample information is made based on whether there is clear evidence for one response over other responses (determined partly by a free parameter controlling how deterministic responding is, similar to a threshold value in sequential sampling models of choice). The EGCM-RT provided a good account for accuracy, mean RT, and the time course data in a variety of categorization tasks, using both separable and integral-dimension stimuli (Lamberts, 2000). Due to the proportional hazards model for feature inclusion and the tight coupling of the stopping rule with accuracy, the EGCM-RT also naturally accounts for RT differences holding across the distribution (i.e, in cumulative distributions, see Maddox et al., 1998).

Kent and Lamberts (2005) subsequently applied the EGCM-RT to absolute identification. Absolute identification has a long history (e.g., Miller, 1956; see Brown et al., 2008, for a review) and as such there are several effects that any model needs to account for. Many models have been suggested (see Stewart et al., 2005, for a review) but only a handful have attempted to account for RTs as well as accuracy. Surprisingly, given the fundamental nature of processes involved in absolute identification, and its continued interest, little consideration had been given to the relation between perceptual processing and performance in absolute identification until the application of the EGCM-RT by Kent and Lamberts (2005). They demonstrated that not only could the EGCM-RT account for the bow effect (response are more accurate and faster for stimuli located at the ends of the range) and set-seize effects (larger set-sizes are less accurately and more slowly responded to than smaller set sizes) it was also able to provide a good account the RT distributions. This extension of the EGCM-RT thus showed that the principles of perceptual information accumulation could be integrated not only into formal models of categorization, but also identification (Kent, 2006, also successfully applied the model to multi-dimensional identification in his unpublished doctoral thesis).

Recently, however, Guest et al. (2010) questioned the significance of the role of perceptual processing in determining free-RT in absolute identification. Nosofsky (1983) reported that stimulus repetitions within a trial in an absolute identification task increased discriminability. Convinced that this was due to increased opportunity for perceptual processing, Guest et al. (2010) completed a variety of experiments designed to determine the underlying cause. To their surprise, the cause was not increased opportunity for perceptual processing, but seemed to be because of increased trial length; forcing participants to respond more slowly by presenting an item multiple times improved performance because it provided more time for response processes to complete. This finding led to a reassessment of the extent of perceptual accumulation processing in the EGCM-RT.

In a recent set of absolute identification tasks, Guest et al. (manuscript in preparation) tried to further elucidate the respective roles of perception, memory, and decision making in absolute identification. Guest et al. (manuscript in preparation) manipulated stimulus exposure duration and found that accuracy increased over exposure duration suggesting gradual information accumulation. However, even at very short stimulus exposures (which offer little time for perceptual processing) it was observed that stimuli near the center of the range were responded to slower than stimuli at the end of the range (the bow effect). Critically, the EGCM normally accounts for an RT bow effect by assuming that for stimuli in the center of the range, responding is less certain (because stimuli have more neighbors and are therefore more confusable) and so more information must be accumulated (resulting in longer RTs). Of course, this is not possible if the stimulus has offset (backward masking was used). To model this finding Guest et al. (manuscript in preparation) assumed that the stimulus sampling mechanism of the EGCM-RT operates in two domains, perceptual processing, which is relatively rapid and drives accuracy, and memory processing, which is relatively slow and drives RTs. Such a model provided a good account for this data. Furthermore, it suggests that future work should focus on disentangling the perceptual and memory sampling processes in other tasks. Our belief is that some tasks involve larger stimulus sampling demands than others. In particular, where the memory representation is weaker, the memory retrieval process is a more significant contributor to RTs than perceptual processing. Nonetheless, it is clear from this work that both information sampling in perception and from memory should be integrated into models of categorization and identification.

## MATCHING AND RECOGNITION

Perhaps the most direct evidence for stochastic sampling of information from stored stimulus representation comes from work on recognition memory. Studies of recognition memory were among the first to benefit from the use of STR methods (e.g., Reed, 1973). Reed (1976) demonstrated that the set size of a list of consonants affected the rate of information retrieval, with a slower rate of retrieval for larger set sizes. Further analysis by McElree and Dosher (1989) on Reed’s (1976) data suggested that the asymptote varied with set size, with smaller set sizes having higher asymptotes. Experiments by McElree and Dosher (1989) also demonstrated that, whilst there was a serial position effect for asymptotic performance and a set size effect on asymptote, the only difference in retrieval dynamics was a faster retrieval rate for items in the last serial position (see also Dosher, 1981). Thus information about items held in memory is clearly retrieved over an extended time period, and the rate at which information can be retrieved is a function of the recency of encoding (a single final item advantage might suggest the item is still held in a relatively accessible state).

Hintzman and Curran (1994) used the STR technique to examine the relationship between judgments of frequency (“How often did this item occur in the study list?”) and old-new recognition. Hintzman and Curran (1994) found that both tasks showed similar information accumulation dynamics, concluding they were driven by a common familiarity process. In two further experiments Hintzman and Curran (1994) looked at the retrieval dynamics of words that were Old, New, and Similar-New (the plural of a singular old word, or vice versa). Hintzman and Curran (1994) found that the false alarm rate (saying “old” to a Similar-New item) initially rose at short signal lags, and then decreased at longer lags. They took non-monotonicity as evidence that, early on in retrieval, a familiarity signal cannot distinguish between Old and Similar-New items, and it is not until a slower recall process (needed to extract the grammatical number of the item) has completed that the Similar-New items can be correctly rejected. However, this dual-process interpretation might not be warranted. If retrieval of (episodic) information linking a specific item to a given context is slower than recall of item information, then the non-monotonicity in Hintzman and Curran’s (1994) data need not be down to separate processes, but instead be the result of different retrieval dynamics for different features of the stored stimulus. Indeed, studies have consistently shown that item information is retrieved faster than associative information (e.g., Gronlund and Ratcliff, 1989; Rotello and Heit, 2000) and position information (Gronlund et al., 1997; see Schneider and Anderson, 2012, for a review).

Brockdorff and Lamberts (2000) developed the feature-sampling theory of recognition (FESTHER) from the EGCM based on the principle that there is considerable overlap between categorization and recognition: recognition requires a decision as to whether an item belongs to one category (“old”) or another category (“new”) based on the similarity to stored exemplars in each category (e.g., Nosofsky, 1988, 1991; Estes, 1994; see Nosofsky et al., 2011, for a recent extension of the ERBW to short-term memory scanning). For Old items it is easy to calculate similarity as the set of studied items is known. However, for New items it is unclear which stored exemplars constitute the relevant comparison group (whether it is all stored exemplars or a subset) and so typically it is assumed that all stored items are used (Estes, 1994; Nosofsky, 1988, 1991). Information is accumulated by element sampling until enough information is available to categorize the stimulus as “old” or “new” based on relative similarity or until a response signal is encountered. Thus the model is formally equivalent to the EGCM for categorization except that the stimulus is compared with the studied items and all stored items.

Brockdorff and Lamberts (2000) applied FESTHER to the data from Hintzman and Curran’s (1994) experiment, which demonstrated the non-monotonic SAT functions for Similar-New items (presented either once or twice at study) and to new data. The initial increase in false alarms was taken as evidence by Hintzman and Curran (1994) as responding due to familiarity and the decrease at longer lags as evidence for an increased use of recall-to-reject. FESTHER, however, predicted the non-monotonicity without the need to include two processes. Instead, according to FESTHER, the initial increase in false alarms for similar items is due to the incomplete stimulus representation leading to a high similarity between Old and Similar-New items. As time increases more features are sampled and the likelihood of sampling the critical feature is increased, resulting in a reduction of false alarms at longer lags (see also Rotello and Heit, 1999). In addition, by assuming the strength in memory of twice-presented Old items is higher than once-presented Old items, FESTHER was able to correctly predict the frequency effect and the tendency for twice-present Similar-New items to have higher false alarms than once-presented Similar-New items at short signals lags.

Brockdorff and Lamberts (2000) further tested the feature sampling account by examining the time course of recognition for individual items in three experiments, by creating a tightly controlled stimulus set (visual objects consisting of 3 or 4 binary valued dimensions). In a study phase a subset of items were shown and participants made feature recognition judgments. At test participants were shown both the original study items (Old) and the unseen items (New). Although this task differs from traditional old-new recognition tasks in that only a limited number of stimuli were used and extensive exposure to each study item stimulus was provided, this afforded the advantage that a detailed analysis of the time course of feature information accumulation could be conducted. FESTHER was able to account for the time course differences between individual items by allowing the perceptual processing rates to vary by feature. Early false alarms for some items were driven by salient dimensions which were processed quickly and made New items perceptually similar to Old items while an increase in false alarms for other items later in processing was caused by feature information that was processed slower making the New items more similar to Old items. Thus FESTHER represents an important challenge to dual process models (although see Göthe and Oberauer, 2008 and Malmberg, 2008, for alternative dual process accounts) but more importantly highlights why it is necessary to consider how perceptual information is processed over the initial stages of recognition in any, single or dual process, model of recognition (e.g., Malmberg, 2008; Schneider and Anderson, 2012).

Although considering perceptual processing is important, clearly there are many other processes operating in cognitive tasks such as recognition. Lamberts et al. (2003) therefore compared the predictions from FESTHER with those from the EBRW when fitting recognition RTs and accuracy for individual stimuli. Both models contained the same assumption regarding computing similarity to all stored exemplars for New items) as both are based on the GCM model of categorization and recognition, e.g., Nosofsky, 1986, 1988). The models differ in the assumed process responsible for differences in choice patterns and RTs with FESTHER focusing of perceptual processing and the EBRW on the time course of retrieval (sampling from memory) and decision making (instantiated by a random walk process). In three free-RT old-new recognition experiments, Lamberts et al. (2003) found the EBRW provided a superior fit to individual stimuli than FESTHER suggesting that in free-RT recognition (although not STR recognition, Brockdorff and Lamberts, 2000) memory sampling and decision making drive a large part of the differences in RTs (see also Ratcliff, 1978; Hockley and Murdock, 1987; Diller et al., 2001; Nosofsky et al., 2011; Schneider and Anderson, 2012, for models which ascribe memory and decision making processes as driving RT in recognition). This may particularly be true when memory traces are weak (as in Lamberts et al., 2003) and need effortful retrieval, but not when stimuli are well learnt (as in Brockdorff and Lamberts, 2000, and in categorization experiments, Lamberts, 2000, 2002). Therefore, the speed at which sampling from memory can be achieved is likely to be a function of practice in a similar way that presentation frequency affects retrieval rates (e.g., Hintzman and Curran, 1994; Brockdorff and Lamberts, 2000) and associative fan (the number of items a cue is associated with) reduces retrieval speed (Schneider and Anderson, 2012). When stimuli are well learnt, and strongly represented in memory, their retrieval times will account for less of the effect on RTs compared with stimuli that have only been seen once or are weakly represented in memory.

In light of the importance of the joint impact of perceptual feature sampling and memory feature sampling in recognition, Lamberts et al. (2002) conducted a set of experiments to examine both processes. They used a variety of simultaneous matching (two stimuli were presented at the same time) and sequential matching (one stimulus was shown first and remained onscreen as the second stimulus was presented) to gain estimates of perceptual processing speeds for the feature on which the stimuli differed. They then used a delayed sequential matching task (analogous to a single item old–new recognition task as there was a 5 s gap between presentation of the first and second stimulus) to examine the rate of feature retrieval. Lamberts et al. (2002) fit data from these tasks using simple feature sampling models for both the perception of features (data from simultaneous and sequential matching tasks) and the joint perception and retrieval of features (data from the delayed-sequential matching task). The crucial finding was that the rate that feature information was retrieved for the stimuli was linearly related to the rate that they were perceptually processed, with feature retrieval taking longer than feature encoding.

Kent and Lamberts (2006a,b) and Lamberts and Kent (2008) further explored the nature of the relationship between the speed at which information about features is sampled in perception and the speed at which information is retrieved about features from stored memory representations. Utilizing the STR method and the sequential and simultaneous matching and delayed matching tasks from Lamberts et al. (2002) but using stimuli built up from three binary-valued dimensions (similar to Brockdorff and Lamberts, 2000). Kent and Lamberts (2006a) were able to estimate the time course of both perception and retrieval for individual features of a stimulus. Kent and Lamberts (2006a) demonstrated that the retrieval rate of features from memory was affected by whether or not the interval between the first and second item of the pair was filled (a simple math’s equation had to be solved) or unfilled (blank screen). The retrieval rates in the unfilled task were faster than the filled task, suggesting the availability of the representation affects the speed of retrieval (assuming the distracter task in the filled condition weakens the encoded representation, similar to a change in serial position, e.g., McElree and Dosher, 1989). However, Kent and Lamberts (2006a) did not find a difference between the retrieval rates of the different features: all features were retrieved at the same speed (unlike the perceptual rates, which varied for all features).

Why did Kent and Lamberts (2006a) not find retrieval rate differences between the different stored features? One reason, mentioned previously, relates to the accessibility of the stored representation, even though a difference was seen between the filled and unfilled conditions, the accessibility of the representation might have still been strong enough not to require complete reconstruction from memory. In order to potentially overcome this problem, Kent and Lamberts (2006b) used a modified form of the matching and delayed-matching tasks, which also more closely equated the demands across tasks. Participants were trained to associate each three binary-valued dimensional object with a unique consonant-vowel-consonant (CVC) label. Participants then carried out four types of task: simultaneous feature-image matching, in which a single feature was presented in isolation next to a complete stimulus image; sequential feature-image matching, simultaneous feature-label matching, in which a single isolated feature was presented next to a CVC label; and sequential feature-label matching. Thus the structure of the tasks involving memory (those involving a CVC label) were identical to the perception only tasks- participants either had to perceive the image or read the CVC and then retrieve the stored representation associated with that label. Across three experiments Kent and Lamberts (2006b) demonstrated robust differences in both perception rates and retrieval rates across the different stimulus features. Generally, there was also a linear relationship between the speed at which a feature was perceived and the speed at which it was retrieved, supporting Lamberts et al. (2002).

However, the tasks used by Kent and Lamberts (2006a) and Lamberts et al. (2002) differ fundamentally from Kent and Lamberts (2006b) in that the latter retrieval tasks can be conceived of as cued-recall and might not involve identical processes as recognition (as in the delayed matching tasks). Lamberts and Kent (2008) therefore reasoned that if the factor driving feature retrieval rate differences was the strength of the representation in memory, then increasing the memory load should weaken the representation, by increasing demand for resources. To test this idea, Lamberts and Kent (2008) manipulated load by presenting either a single item or two items to be remembered in a delayed matching task. This made it less likely that the information could be held continuously in a rapidly accessible form, and thus increasing the need to retrieve the representation from a more durable longer-term store (this conception is consistent with the focus of attention models of memory by Cowan, 2001; Garavan, 1998; Oberauer, 2002). The data showed feature retrieval rates varied in the two-item delayed matching task with a linear relationship between the rate of feature processing and feature retrieval (supporting Lamberts et al., 2002, and Kent and Lamberts, 2006b) but no differences in feature retrieval rates in the one-item delayed matching task (replicating Kent and Lamberts, 2006a). Retrieval demands (based on the strength of the representation in memory) appear to at least partially determine whether or not features vary in how quickly information can be retrieved about them. If the representation is strong (it is in the current focus of attention) then retrieval is fast for all features, however, if the representation is weak (not in the current focus of attention) then retrieval is more effortful (for example by reconstructing the stimulus back into the focus of attention).

The ability of a simple stochastic feature sampling process to predict both perception and retrieval of stimulus feature information, and the close relationship between the speed of perception and retrieval, led Kent and Lamberts (2008) to suggest that the link between the time course of encoding and the time course of retrieval arises because, in order for information to be retrieved, a quasi-perceptual reinstatement of the initial encoding event must take place (which is not needed if the representation is already held in the focus of attention). In order to reactivate a stored representation similar neural pathways are used in a mental simulation of the perception of that stimulus. Although this theoretical interpretation is not necessary, it links the task of matching and recognition (and therefore categorization) to a broader cognitive architecture based on mental simulation (e.g., Barsalou, 2008); we discuss the importance of this in the Section “Future Directions.”

## VISUAL ATTENTION AND SEARCH

Examining perceptual processing using STR procedures has a long and productive history in visual cognition research (e.g., early work by Eriksen and Schultz, 1979). For example, STR procedures have been used to examine the influence of visual attention on discrimination and the rate of perceptual processing. Although the influence of visual attention on discrimination has been established for some time (e.g., Posner, 1980; see Carrasco, 2011, for a recent review) in order to disentangle the effect of attention on discrimination and on processing speed an STR procedure is required. Using this procedure Carrasco and McElree (2001) showed that cueing a target location accelerates the rate of visual information processing (see also Liu et al., 2009). This benefit of attention is observed using exogenous and endogenous cues, although cue validity modulates this benefit for endogenous cues only (Giordano et al., 2009). More recently, the STR approach has also been used to demonstrate that temporal preparation results in an earlier onset of visual processing (Bausenhart et al., 2010). Unpublished work from our laboratory has demonstrated that, in perceptual categorization, visual attention can modulate feature processing rates independently of perceptual salience (Lamberts and Kent, 2006, unpublished manuscript) or feature diagnosticity (Guest and Lamberts, 2008). Thus STR work on visual attention exemplifies the usefulness of the basic approach.

One of the central fields of visual cognition research in which information accumulation models have been used is that of visual search. Historically, an issue of conjecture in visual search is the difference between feature search (search for a target that has a unique value on one of its features such as a blue T amongst yellow Ls and Ts) and conjunction search (search for a target uniquely defined by a conjunction of features, such as a blue T amongst blue Ls and yellow Ts). Some models of search have argued that different processes are involved in these search tasks (e.g., Treisman and Gelade, 1980; Wolfe, 1994): feature search is pre-attentive because the target “pops” out of the display due to its unique feature whereas in conjunction search the target has no unique feature and so search involves serial shifting of attention from item to item. Others have suggested that both feature and conjunction search can be explained by a limited capacity parallel process. In this context, a number of studies have examined whether adding additional distractors to a search task influences the rate of processing in feature and conjunction search tasks through the use of STR methods. McElree and Carrasco (1999; Carrasco and McElree, 2001; Carrasco et al., 2006) showed that increasing set size decreased visual information processing rates in conjunction search but not feature search. Although at face value this appears to support the notion of different processing mechanisms underlying feature and conjunction search, fitting information accumulation models to the time course data, which assumed either parallel or serial sampling, indicated that performance in both feature and conjunction search could best be explained by a limited capacity parallel process (see also Dosher et al., 2004, 2010). Indeed, a reanalysis of Carrasco and McElree (2001) data, alongside further experimentation, showed that increasing display size from one item to more than one item does influence processing rates in feature search, suggesting visual information processing is still capacity limited in feature search (Kent et al., 2012). Kent et al. (2012) also demonstrated that information processing rates were affected by the stimulus duration, with a longer duration speeding processing rates when distractors were present, but not when distractors were absent. Kent et al. (2012) interpreted this as indicating that at shorter durations the stimulus representation from which discrimination took place was noisier than when stimulus exposure duration was longer (see also Liu et al., 2009; Smith and Sewell, 2013 for a similar argument).

Building on this previous work which examined the rate at which search displays were processed (e.g., Carrasco and McElree, 2001), Guest and Lamberts (2011) developed a model of visual search based on the principles of the EGCM (the EGCM-VS) that specifies the processing of component features of individual display items. The EGCM-VS assumes that information about each item and about each item’s features is processed independently and in parallel. Each item’s representation is then compared to the representation of the target in memory. The probability of a target present response is based on the combined overall similarity of display items to the target and the bias toward making a target absent response. The EGCM-VS is unique in that it explicitly claims that similarity relationships in a display are dynamic and change over time as perceptual information is accumulated about stimulus features. Thus the salience of the features that make up the display items is crucial in explaining how visual search performance changes over time. As with perceptual categorization, this dynamic similarity perspective enables the model to account for non-monotonic changes in response accuracy with increasing display duration, which models that assume static time invariant similarity have difficulty dealing with. Guest and Lamberts (2011) showed that the model could account for the time course of performance in a wide variety of search tasks including feature search, conjunction search, triple conjunction search and search displays with different ratios of homogeneous distractors. Moreover, by modeling perceptual processing of object features the EGCM-VS enables examination of how feature processing is influenced by characteristics of the display such as distractor homogeneity, which appears to accelerate the rate of feature processing. Of course, other models of visual search such as guided search (Wolfe, 1994), signal detection models (e.g., Eckstein, 1998; Palmer et al., 2000), optimal models (Ma et al., 2011) and models related to the theory of visual attention (Bundesen, 1990; Logan, 2002), could feasibly integrate a formal mechanism for describing the accumulation of feature information and this is a challenge for future research. One promising model of visual attention developed by Ratcliff and Smith (2009) and applied to multi-element displays by Smith and Sewell (2013) includes a detailed description of how sensory information is transferred into VSTM. The process of building a representation in VSTM includes the accumulation of information from a sensory trace (similar to Busey and Loftus, 1994) which then feeds a diffusion process for response generation. Although it is conceivable that the construction of the VSTM trace is associated with different information accumulation rates (by allowing the attention gain function to vary not only due to whether an item is attended or not, but by the different features of each item), it is not clear currently how the model could be adapted to account for tasks in which the different features of a stimulus are more or less relevant, without a fundamental change to the core assumptions.

## WORD IDENTIFICATION

One form of stimulus that is clearly built up of constituent features is the written word. Each individual letter can be considered a feature of the word just as each letter itself has features (e.g., Bower, 1967). Whilst some of the early research on information accumulation was based on letter string stimuli (e.g., Rumelhart, 1970), interest in this approach to word-like stimuli dwindled in favor of approaches that implied a static similarity structure among stimuli, that is, similarity between stimuli did not change as a function of processing time. The most notable of these was the Interactive Activation approach (McClelland and Rumelhart, 1981; Rumelhart and McClelland, 1982).

The Interactive Activation framework involves the calculation of a balance of facilitatory matching information and inhibitory or discriminatory mismatching information for any given node (feature detector); node activation is driven by this net input to an ideal value; these net inputs operate as unnormalized similarity scores that ramp up proportionally with input strength, leaving normalized similarity unchanged over time. Even with the introduction of noise (McClelland, 1991), changes in activity represent a smooth and relentless march toward the correct option. Contemporary competitor models included some that were even more explicitly based on static similarity calculations, using transformations of confusion probabilities to calculate activation (Paap et al., 1982).

Alongside the focus in other areas on integrating feature sampling into models of cognition (e.g., Lamberts, 2000), two types of challenge to the manner in which these word recognition models worked set the stage for the recent re-introduction of the idea of information accumulation to visual word recognition: renewed arguments for left to right processing of letters, and evidence relating to the way in which anagrams of words (“wrods”) are perceived.

It is obvious that in written languages that transcribe spoken language from left to right, reading proceeds word by word in a broadly left-to-right sequence. Moreover, within a word, graphemes transcribe from left to right phonemes that are said in temporal sequence, and recognition of spoken words indeed proceeds on an initial (incomplete) stimulus (Marslen-Wilson, 1984). It therefore seems natural that letters might be accumulated in visual word perception in a strictly left to right sequence. Among the evidence that spoken word recognition works on an initial portion of the stimulus are uniqueness point effects: advantages in the recognition of words whose identity can be inferred from a few initial phonemes due to the lack of competitors. Kwantes and Mewhort (1999) and Lindell et al. (2003) ran analogous studies comparing words for which the left-most three or four letters uniquely identified the word (e.g., ACTRESS) with those for which more letters from the left, six or seven (e.g., ABSOLVE) would be required. Indeed, the former items were named and given lexical decisions more rapidly, which would be expected if letter processing proceeded left to right, and lexical access could begin once a unique word was isolated by the available letters. However, Lamberts (2005) showed that these items also differed in confusability without special consideration to left to right processing. Simulations in which the letters were processed in random order, and lexical access began once a unique word was isolated by the available letters predicted the same effect.

Nevertheless, theorists such as Whitney (2001) have pointed to other phenomena as indicating a left-to-right process, such as a left-to-right gradient in accuracy of identification of letters in briefly presented strings. Indeed, Whitney has claimed that a reliable temporal lag (on the order of 10 ms or greater) between letters in left-to-right sequence is critical to the correct identification of letter order, which is a major contemporary issue in visual word recognition (Grainger, 2008). Adelman et al. (2010) examined whether this was the case by manipulating the duration of the stimulus in 6 ms increments in a two-alternative forced choice task on four-letter words. Whilst differences in accuracy emerged, this could not be attributed to a lag in processing (and certainly not one of 10 ms/letter magnitude) because for all letter positions, accuracy was at chance for 18 ms presentation and above chance for 24 ms presentation. Moreover, performance on pairs like CART–CAST was worse than those like CART–CAMP, despite the fourth letter being irrelevant to performance under a fully left to right account (Adelman, 2011, and unpublished data from Adelman et al., 2010). An information accumulation account like that simulated by Lamberts (2005) of course can account for these patterns with only the assumption that processing is more efficient (higher processing speed) for letters to the left.

Indeed, Adelman’s (2011) Letters in Time and Retinotopic Space (LTRS) model was built on this idea to produce an account that includes the processing of letter order. A variety of findings have pointed to the fact that letter position is either not used precisely in the processing of letter strings (ROGUE and ROUGE are confusable), which requires that models do not use a simple slot based system where letters that appear in (for example) second position are only compared to the second letter of known words. For the identification of strings, in LTRS, information accumulation accounts for the difficulty of anagrams by assuming that both letters must be perceived to know their relative order: either “W” or “A” may be perceived to know SWAN is not STUN, but both “W” and “A” must be perceived to know SWAN is not SAWN (in fact in the model it is alternatively possible to perceive that “W” is adjacent to “S” or “A” to “N,” but this is a slower process).

This implies a non-static similarity process in which stimuli pass from being matches to known words to being mismatches. Such a process contrasts with models in which stimuli produce match scores to known words (e.g., Grainger and van Heuven, 2003; Davis, 2010), or behavior stems from the distance between words and letter strings in psychological space, with percepts being noisy samples of locations in that space (Norris and Kinoshita, 2012). This is seen in their explanation of the most commonly used paradigm, masked form priming, in which a brief (ca. 50 ms) prime (e.g., “wlaker”) precedes a clearly presented target (e.g., “WALKER”) which requires a response (typically lexical decision); responses are faster when primes are similar to targets. In other models, the partial match between prime and target persists throughout the prime’s presentation, and it thus evokes an attenuated target-like response. For example, “wlaker” activates a unit for “WALKER,” but not as much as “walker” would; or on each (and every) time step, the noisy sample of “wlaker” will probably have a relatively good likelihood of having been produced by a stimulus “WALKER,” at least compared to control.

The Letters in Time and Retinotopic Space model offers a simple feature sampling account relying on dynamic match-mismatch similarity. It assumes primes activate targets without attenuation, but target activation stops increasing (but does persist) once the prime no longer matches the target; target-like processing of the prime is truncated not attenuated. For primes with few features in common with the target, a mismatching feature is usually perceived early producing little priming; for primes with many features in common, a mismatching feature comes much later. When order is involved, more than one feature must be perceived to produce the mismatch, making anagram primes (if the distortion is minor, e.g., “wlaker”) particularly effective. Through re-integrating the notion of stochastic feature sampling into word identification and word priming research, LTRS demonstrates the importance of considering how perceptual processing at the level of visual feature influences word reading.

## FUTURE DIRECTIONS

In this review, we have tried to demonstrate why integrating feature sampling into models of cognitive processes is important. Our central argument is that, in a wealth of cognitive tasks, the time taken for perceptual processing can be a large proportion of the time taken to complete these tasks. Moreover, because in everyday life and in the lab, time for processing can be short, decisions are often made using only incomplete perceptual and memory representations. Although seemingly obvious, this point is important for several reasons. As noted above, re-evaluating tasks from a feature-based information accumulation perspective can show that previous findings, such as in word identification (Adelman, 2011) and recognition (Brockdorff and Lamberts, 2000), may, in part, be explained through consideration of perceptual processes. Exploring the time course of performance also reveals patterns of data, such as non-monotonic changes in response accuracy with increasing stimulus duration (e.g., Lamberts and Freeman, 1999a; Brockdorff and Lamberts, 2000; Guest and Lamberts, 2011) that can be readily explained by feature sampling models, but presents challenges for models without a feature sampling perceptual processing component. Finally, by arguing for the importance of feature sampling in cognition we hope to highlight the need to consider the role of perceptual processing in cognitive tasks, even where the perceptual component may appear relatively minor. A recent example comes from Inglis and Gilmore (2013) who noted that in studies of the approximate number system (ANS) estimates of ANS acuity varied between studies but so did the duration for which stimuli were presented. Ingles and Gilmore demonstrated that differences in the acuity of ANS representations with changes in stimulus duration could be best described by a perceptual processing information accumulation model. We believe that evaluation of perceptual processing mechanisms is therefore a useful and important endeavor.

It is now clear that it is important to consider both feature sampling form the sensory store and representations held in memory. Kent and Lamberts (2008) have argued that sampling from the display and sampling from memory involve closely related and overlapping processes. This observation fits in well with the notion that cognition is grounded and that the same perceptual-action systems involved in stimulus encoding are also partly involved in retrieval (e.g., Barsalou, 2008). Clearly, more work is needed in developing a detailed computational account of grounded cognition (Pezzulo et al., 2013); we suggest that categorization, identification, and recognition might provide an excellent test bed as indeed they have in the past (e.g., Nosofsky, 1992). However, the link between encoding and retrieval also raises a number of, as yet unanswered, questions. An important question is what is the main driver of the time course of performance in the tasks we have reviewed? Is it sampling from the display or is it sampling from memory? We think the answer will undoubtedly be complex and will depend on a number of components including: stimulus-driven factors, such as the complexity or number of items in the display, the complexity and discriminability of object features; process limitations, such as limited attentional resources; memory factors, such the strength of representations and the number of relevant comparison items in memory; and task-based demands, such as the category structure and the number of response options. As a first step toward exploring these issues we have begun to formalize the relationship between the perceptual and memory sampling processes in a forthcoming article (Guest et al., manuscript in preparation). In our model of absolute identification, memory sampling and matching begins as soon as a perceptual information element has been processed. Estimates of the length of the different sampling processes can be estimated, in this task yielding a short perceptual sampling process followed by a longer memory sampling process. Importantly, in this task the perceptual component is simple (involving a single dimension) and the memory component more complex (comparison of the stimulus to multiple, highly confusable, stored representations). In comparison, in visual search, there are many display items that need to be encoded and compared with a single stored representation (the target). Such key differences might well modulate the relative roles of the perceptual and memory sampling processes in determining task performance.

Whereas models based around the simple processing assumptions of the EGCM have had some success, it is clear that the model itself is simplistic and will need fleshing out before it can be considered a complete process model of perceptual cognition. An alternative approach is to take existing successful models of perceptual decision making and augment them with a stochastic feature sampling mechanism (e.g., Lamberts, 2002). For instance, Biederman et al. (1999) cite the evidence of Lamberts (1998) as support for their theoretical assumption of featural representations (Hummel and Biederman, 1992). Indeed, many of the most compelling paradoxes of decision making come from multiattribute choice (e.g., Tversky, 1972); the stochastic nature of feature processing for the different attributes might be important in generating the pardoxes. Already many recent models include processes of information accumulation (e.g., see Logan, 2004; Ratcliff and Smith, 2004, 2009; Purcell et al., 2010; Smith and Sewell, 2013). However, it is non-trivial to integrate separable feature stimuli into these models; it is, nonetheless, an important and worthwhile endeavor for future research.

It is also important to integrate the reviewed mechanisms for feature and memory sampling into a neurobiological framework. In categorization, the COVIS model developed by Ashby et al. (1998, 2011) is a good example of this approach. In COVIS, category learning involves multiple systems that are localized in different brain regions (Ashby et al., 2003; Filoteo et al., 2005; Maddox and Filoteo, 2005) with an implicit procedural-based system that mediates category learning when it is necessary to integrate information from multiple dimensions and an explicit hypothesis-testing system that mediates rule-based category learning. Although offering a potential framework within which to explore perceptual processing mechanisms in categorization, the notion of multiple systems has been repeatedly questioned (Gureckis et al., 2011; Newell et al., 2011; Dunn et al., 2012).

In terms of the biological basis for feature and memory sampling, evidence from studies with monkeys (for a review see Gold and Shadlen, 2007) and humans (for a review see Heekeren et al., 2008) suggests multiple neural systems mediating human perceptual decision making. Heekeren et al. (2008) suggest four distinct systems. In the first, lower level sensory regions are involved in accumulation of sensory evidence, the exact region depending on the task (e.g., the fusiform face area and the parahippocampal place area in a face-house discrimination task). At higher levels, such as the dorsolateral prefrontal cortex, this sensory evidence is integrated and compared in order to compute a decision, with activity in such areas being likened to a diffusion process (Schall, 2001; Gold and Shadlen, 2007) It seems probable then that feature and memory sampling are mediated by these two systems, respectively. A further system, involving areas such as the anterior insula and the inferior frontal gyrus, is thought to detect perceptual difficulty and signal when more resources are required (e.g., attention). Such a system could well be involved in determining the extent of feature and memory sampling required. This will also depend on the speed-accuracy tradeoff, which seems to be modulated by the pre-supplementary motor area (Bogacz et al., 2010). A final system involving areas such as the posterior medial prefrontal cortex is thought to monitor performance and adjust decision strategies to maximize performance. This system may determine trial to trial differences in the processing and utilization of feature information. Substantial progress has therefore been made in determining the neural systems mediating perceptual decision making, and relations between these systems and components of the feature sampling account are apparent. An important avenue for future research is to explore the evidence for such links and work toward development of a computational cognitive neuroscience approach in this area.

Individual and group differences are also an increasingly important aspect to be considered in basic cognitive research (e.g., Kanai and Rees, 2011). It is clear people have different SAT curves, and it is also likely that people will vary in the relative rate at which they complete cognitive tasks, including simple feature perception (e.g., Salthouse, 1996). As an illustrative example, Guest et al. (manuscript in preparation) recently conducted a series of experiments which showed age related slowing in visual information processing speed for tasks requiring visual search and processing and maintenance of multiple items in visual working memory. Moreover, comparison of processing rates between tasks indicated that maintaining multiple item representations led to a more age related decline visual information processing rates than a search task in which multiple distractors were dismissed online. Thus, understanding how the temporal dynamics of cognition changes across the lifespan and varies between individuals could provide a rich vein of data about individual differences in cognition. Such research should investigate these differences within the context of formal models in order to better understand the processes underlying individual differences.

Although we make the case for considering the role of perceptual processes in cognitive tasks, we appreciate that this is not without additional complications. Typically, in order to study the time course of information accumulation, a STR procedure is required, which: increases the number of required trials (often by a factor of at least 5–7, and hence the duration and cost of an experiment by the same factor); introduces additional cognitive load into the task which participants find unnatural to complete; requires extensive training, potentially altering the sample of participants who can complete the task and the strategies they use (having already had extensive experience on the task); and the loss of many data points. Although these issues should not preclude the use of STR designs, and measures can be taken to mitigate their impact, these are clearly a disincentive for using such designs. Recently, Kent et al. (2014) presented data on the use of mouse tracking as an alternative to the STR procedure (see also Spivey et al., 2005; Dale et al., 2007; Freeman, 2014). Mouse tracking (recording the X and Y position over time toward response options), or indeed other forms of dynamic responding (e.g., reaching movements, Song and Nakayama, 2009), provides an advantage in that the response is natural, not time restricted, and involves little or no practice. By requiring participants to attempt to start making their response immediately after stimulus onset, we argue it is possible to measure early choice preferences before a complete stimulus representation has been formed. Clearly further work needs to be undertaken before the relationship between STR data and dynamic response tracking data is fully appreciated (e.g., Friedman et al., 2013), but for now we note that we have replicated both the early category cross over effects reported by Lamberts (1995; Lamberts and Brockdorff, 1997; Lamberts and Freeman, 1999a; Guest and Lamberts, 2011) and the rate differences in discrimination experiments (Carrasco and McElree, 2001). It is hoped that dynamic response techniques may make it much easier to elucidate the perceptual mechanisms in cognitive tasks, enabling greater focus on how the time course of perceptual processing influences performance in a much broader range of cognitive tasks. Work in other others is also set to benefit from these insights, for example, Freeman (2014; see also Freeman and Ambady, 2011; Freeman et al., 2011) has attempted to demonstrate early implicit attitudes (such as gender stereotypes) at least partly reflect the early dominance of more salient dimensions of faces (e.g., hair length).

The idea that information is accumulated gradually, feature-by-feature, from a stimulus has a long history, and it is clear from recent developments in a number of core cognitive areas, and more recently social cognition, that the need to understand this process and incorporate it in models of both perception and memory is important to understanding how people make decisions based on partially constructed stimulus representations.

## Conflict of Interest Statement

The authors declare that the research was conducted in the absence of any commercial or financial relationships that could be construed as a potential conflict of interest.
